# Systematic characterization of SARS-CoV-2 spike protein subunit trafficking and secretion reveals enhanced strategies for vaccine design and quantification

**DOI:** 10.3389/fmicb.2026.1856886

**Published:** 2026-07-27

**Authors:** Qian Zhang, Maria Hammond, Jie Song, Zongwei Fang, Hongxing Zhao, Maréne Landström, Ya-Fang Mei

**Affiliations:** 1 Department of Clinical Microbiology, Umeå University, Umeå, Sweden; 2 Umeå Center for Microbial Research (UCMR), Umeå University, Umeå, Sweden; 3 Department of Immunology, Genetics, Pathology, SciLifeLab; Proximity Proteomics Facility, Uppsala, Sweden; 4 Department of Medical Bioscience, Umeå University, Umeå, Sweden

**Keywords:** antigenicity, characterization, PEA quantification, SARS-CoV-2, secretion, spike and subunits, trafficking, vaccine candidate

## Abstract

Despite the widespread deployment of current COVID-19 vaccines, significant gaps remain in understanding the complete biological behavior of the SARS-CoV-2 spike (S) protein. Through systematic characterization of mammalian expression systems, this study shows that the full-length S protein exhibits a complex intracellular distribution, predominantly localizing not only to the cell membrane but also to the cytoplasm, nucleus, and extracellular compartments. Comparative analyses revealed distinct subunit-specific trafficking patterns. The S1 subunit showed increased intracellular accumulation and secretion compared to the full-length S protein, although with reduced surface expression. Conversely, the receptor-binding domain (RBD) and S2 domains were mainly associated with cytoskeletal (CS) structures. Notably, the signal sequence-enhanced RBD (SS-RBD) construct engineered in this study demonstrated dramatically enhanced extracellular accumulation, approximately 100-fold higher than the full-length S protein and 10-fold greater than S1, as measured by proximity extension assay (PEA). Signal peptide modification effectively redirected RBD from CS retention to efficient secretion, significantly improving detection sensitivity. PEA outperformed conventional methods such as flow cytometry (FACS), Western blotting (WB), and immunofluorescence, offering sensitivities several orders of magnitude higher. Consequently, these findings provide: (1) a structural framework for rational antigen design by distinguishing essential versus dispensable domains; (2) experimental support for SS-RBD as a promising vaccine candidate due to its high secretion efficiency and preservation of neutralizing epitopes; and (3) a robust platform using PEA for high-sensitivity antigen characterization. This study enhances the fundamental understanding of spike protein biology and offers actionable insights for developing next-generation vaccines targeting SARS-CoV-2 and related coronaviruses.

## Introduction

Coronavirus disease 2019 (COVID-19), caused by severe acute respiratory syndrome coronavirus 2 (SARS-CoV-2), continues to exert profound effects on global public health, economic stability, and social systems. Since the onset of the pandemic, unprecedented efforts have been directed toward developing effective vaccines. However, the persistent emergence of viral variants underscores the need for robust, adaptable vaccine platforms.

The SARS-CoV-2 spike protein is a major structural and antigenic component that plays a central role in viral entry and immune recognition. It is a heavily glycosylated trimer, with each protomer comprising 1,273 amino acids. Cleavage at position 685 separates the protein into subunit 1 (S1) and subunit 2 (S2) (Watanabe et al., 2020; V'kovski et al., 2021). The S1 subunit contains the N-terminal domain (NTD) and the RBD. In contrast, the S2 subunit (residues 686–1,273) includes the fusion peptide (FP), heptad repeats (HR1 and HR2), transmembrane domain (TM), and cytoplasmic tail (CT) (Wrapp et al., 2020). Structural studies have resolved the interaction between the RBD and the host receptor angiotensin-converting enzyme 2 (ACE2), and numerous neutralizing antibodies (NAbs) have been shown to target this interface, thereby blocking viral entry (Wang et al., 2020). Additional NAbs recognizing regions outside the receptor-binding motif (RBM) further underscore the complex antigenic landscape of the spike protein (Ju et al., 2020). This multi-domain architecture highlights the importance of structure-guided antigen design.

Genetically engineered subunit vaccines targeting the spike protein, such as those developed by Novavax, have demonstrated efficacy and safety in clinical trials (Follmann et al., 2024). However, their reliance on yeast-based expression systems presents inherent limitations. Although yeast platforms enable high antigen yield, mammalian expression systems more closely replicate native post-translational modifications and protein folding, which are critical for preserving antigenicity and immunological relevance. To address these challenges, this study investigates spike-derived subunit antigens expressed in mammalian cells, aiming to combine accurate post-translational processing with enhanced secretion efficiency to support improved vaccine design.

To characterize protein expression and distribution, multiple analytical approaches were used, including flow cytometry (FACS), WB, immunofluorescence (IF), and the proximity extension assay (PEA). PEA is a highly sensitive immunoassay that enables the detection of low-abundance proteins in complex biological samples (Lundberg et al., 2011). It utilizes pairs of antibodies conjugated to unique DNA oligonucleotides; upon target binding, the DNA strands are brought into proximity, allowing hybridization and extension by DNA polymerase to generate a quantifiable sequence. This sequence is subsequently amplified using real-time PCR or next-generation sequencing, providing a precise measurement of protein abundance. PEA has been widely applied in biomarker discovery and clinical research, including recent studies demonstrating high sensitivity and specificity in detecting SARS-CoV-2-related antibodies (Zhao et al., 2022).

Despite extensive structural characterization of the spike protein, its intracellular behavior in mammalian cells remains incompletely understood. The continued emergence of SARS-CoV-2 variants with mutations in the spike protein further emphasizing the need to understand how structural differences influence protein localization, trafficking, and antigenicity (Wang et al., 2021; Ou et al., 2022). While earlier studies, including previous studies by the authors, have examined aspects of spike protein localization, a systematic and comparative analysis is still lacking (Zhang et al., 2020; Jiang and Mei, 2021).

This study comprehensively investigates the expression, subcellular distribution, and secretion profiles of the full-length spike protein and its subunits in mammalian cells. Furthermore, this study evaluates the immunological and structural properties of spike protein to identify candidate antigens with improved characteristics for vaccine development. Specifically, three critical gaps are addressed: (1) comparative secretion efficiency of spike subunits, (2) subcellular localization patterns, and (3) structural features with potential antigenic implications, with a specific PEA detection method.

## Materials and methods

### Construction of recombinant spike protein and its subunits

The SARS-CoV-2 spike protein sequence was retrieved from the GenBank database (accession number: MN908947). Codon-optimized cDNA sequences encoding the full-length spike protein and its selected subunits were synthesized and cloned into the mammalian expression vector pUC57, incorporating a C-terminal 6×His tag for purification and detection purposes. The S1 subunit was also generated from the original cDNA sequence without codon optimization and studied in parallel, designated S1-ori.

### Cell lines and antibodies

HEK293 cells were purchased from Microbix Biosystems Inc. (Toronto, Canada), and A549 cells were provided by Walter Nelson-Rees (University of California, Berkeley, United States). Cell culture and passage of HEK293 and A549 cells were performed as previously described (Mei et al., 2016). A rabbit anti-SARS-CoV-2 neutralizing antibody (NAb) (clone 4G6) was obtained from Genscript (Cat. No. A02053-100). Rabbit monoclonal antibodies against 6×His-tags (12698S) were purchased from Cell Signaling Technology. The following antibodies were obtained from Abcam: rabbit monoclonal anti-histone H2A.X (ab124781), calreticulin ER marker (ab92516), cytoskeleton marker cytokeratin 18 (ab133263), *β*-tubulin antibody (ab6046), and goat anti-rabbit IgG H&L HRP-conjugated secondary antibody (ab207718). A FITC-conjugated polyclonal swine anti-rabbit immunoglobulin antibody was purchased from DAKO, and a non-conjugated normal rabbit IgG control antibody was obtained from Santa Cruz Biotechnology (sc-3888).

### FACS assay

For kinetics experiments, 3 × 10^5^ cells were seeded in 12-well plates and cultured overnight. The following day, cells were transfected with spike protein expression plasmids. According to the manufacturer’s instructions, either 1.6 μg or 1.1 μg of plasmid DNA per well was mixed with 4 μL of lipofectamine 2000 and incubated for 24, 48, or 72 h. Post-transfection, cells were detached using PBS containing EDTA, centrifuged at 800 rpm for 5 min, and washed once with PBS supplemented with 1% bovine serum albumin (BSA) (BP buffer). Cells were then fixed in 100 μL of 4% paraformaldehyde for 20 min at room temperature (RT), followed by one wash in BP buffer at 4 °C. For intracellular staining, cells were permeabilized and blocked in PBS containing 1% BSA and 0.1% saponin for 30 min. After blocking, cells were incubated with a rabbit anti-spike protein polyclonal NAb (1:1000 dilution in BP buffer) for 1 h at 4 °C with gentle shaking. Cells were then washed and incubated with FITC-conjugated polyclonal swine anti-rabbit IgG antibody (DAKO; 1:40 dilution in BP buffer) for 1 h on ice with shaking. After a final wash, cells were analyzed by FACS using a Bio-Rad ZE5 instrument (United States). Data analysis was performed using FlowJo VX software (EXCITE, EXPERT Cytometry; design: BKSURU.com, United States). To account for possible masking of the RBD due to ACE2 interaction, a rabbit monoclonal anti-6×His tag antibody was also used to detect RBD expression in transfected cells. Cells transfected with an empty vector served as negative controls.

### Cell lysate, pellet, and supernatant preparation

Culture media in 1,500 μL from transfected cells were collected at 24-, 48-, and 72-h post-transfection (hrs.p.t.) and centrifuged at 1500 rpm for 5 min to pellet the cells. The supernatant was carefully collected and immediately stored at −80 °C. The cell pellets were washed once with cold PBS and then used for downstream analysis or lysate preparation. Cell lysates and pellets were prepared following the Bio-Rad protocol (available at: www.biorad.com/webroot/web/pdf/lsr/literature/Bulletin_6376.pdf). Cell lysis samples in 100 μL were stored at −80 °C until further use. Protein concentration in the cell lysate was determined using the BCA assay (Pierce BCA Protein Assay Kit, Thermo Scientific, United States). The cell pellets were resuspended in 40 μL of double-distilled water (ddH₂O) and gently mixed. Both PEA and immunoblot assays were performed to assess the presence and levels of full-length spike protein and its subunits in the cell supernatant.

### SDS–PAGE and immunoblot assay

SDS–PAGE and WB were performed to detect SARS-CoV-2 spike protein and its subunits in cell lysates, pellets, and supernatants. Sample loading included 30 μL of supernatant, 10 μg of total protein from the cell lysate, and 5 μL of resuspended cell pellet solution per well. Proteins were separated using NuPAGE SDS–PAGE gels and transferred to nitrocellulose (NC) membranes following the NuPAGE Technical Guide protocol with minor modifications.^1^ Roche Diagnostics, Germany in TBST buffer (1 × Tris-buffered saline, 0.1% Tween-20, pH 7.4) for 1 h at room temperature. Membranes were then incubated overnight at 4 °C with rabbit anti-His-tag primary antibodies (1:2,000 dilution). After washing, membranes were incubated with horseradish peroxidase (HRP)-conjugated goat anti-rabbit IgG secondary antibodies (Abcam; 1:10,000 dilution) for 1 h at room temperature. Detection was performed with an enhanced chemiluminescence (ECL) reagent (Thermo Fisher Scientific, United States), and signals were visualized on CL-XPosure film (Thermo Fisher Scientific, United States). Exposure times ranged from 1 s to 30 min, depending on signal intensity.

### Immunoblotting for subcellular fractionated proteins

A total of 1 × 10^6^ cells were seeded in a six-well plate and transfected with five different SARS-CoV-2 spike protein fragment constructs. At 48 h post-transfection (p.t.), cells were subjected to subcellular fractionation using the Subcellular Protein Fractionation Kit (Thermo Scientific, Cat. No. 78840) according to the manufacturer’s protocol. Protein concentrations of the eluted subcellular fractions were quantified using the Pierce™ BCA Protein Assay Kit (Thermo Fisher Scientific, Cat. No. 23227).

Equal amounts of protein (10 μg/lane) were used for SDS-PAGE analysis, except for the cytoskeletal (CS) fraction, for which 5 μg was loaded due to low yield. Proteins were separated by SDS-PAGE and transferred to NC membranes. WB was performed using antibodies specific to each subcellular compartment and the spike protein subunits. The primary antibodies included: calreticulin (ER marker; Abcam, ab92516) to detect membrane (M) and endoplasmic reticulum (SN) fractions; *β*-tubulin (Abcam, ab6046) for the cytoplasmic (CP) fraction; cytokeratin 18 (cytoskeleton marker; Abcam, ab133263) for the soluble nuclear (SN) and CS fractions; histone H2A.X (rabbit monoclonal; Abcam, ab124781) for the chromatin-bound nuclear (CBN) fraction; anti-6×His tag (rabbit monoclonal; Cell Signaling Technology, 12698S) to detect spike protein subunits. Membranes were incubated with primary antibodies at a dilution of 1:1000, followed by incubation with HRP-conjugated goat anti-rabbit IgG secondary antibodies (Abcam, 1:10000 dilution). Protein bands were visualized using Pierce™ ECL Western Blotting Substrate (Thermo Fisher Scientific, Cat. No. 32106).

### Immunofluorescence and confocal microscopy

HEK293 and A549 cells were seeded at a density of 80,000 cells per well on 12-mm glass coverslips in a 24-well plate and incubated overnight at 37 °C. The following day, cells were transfected with Lipofectamine 2000 and five spike protein expression plasmids, or an empty vector as a control, and incubated for 24 h. Post-transfection, cells were washed with phosphate-buffered saline (PBS) and fixed with 4% paraformaldehyde (PFA) in PBS for 20 min at room temperature (RT). After extensive PBS washing, the cells were permeabilized with 0.5% Triton X-100 in PBS for 10 min. Permeabilized cells were then blocked with 5% bovine serum albumin (BSA) in PBS before incubation with rabbit monoclonal anti-6×His tag primary antibody (Cell Signaling Technology) diluted in 1% BSA-PBS. Primary antibody incubation was carried out overnight at 4 °C. Following primary labeling, cells were washed and incubated with FITC-conjugated swine anti-rabbit immunoglobulin secondary polyclonal antibody (DAKO) diluted 1:40 in 1% BSA-PBS for 1 h at RT. Nuclear staining was performed with 4′,6-diamidino-2-phenylindole (DAPI) for 1 min at RT, followed by extensive washing in PBS. Coverslips were mounted onto microscope slides using Dako Fluorescence Mounting Medium (Agilent). Imaging was acquired using a Carl Zeiss LSM 710 confocal microscope equipped with a 63 × oil immersion objective lens (numerical aperture 1.4). Image acquisition and processing were conducted using Zen 2011 software. All image scoring and analysis were performed under blinded conditions.

### Proximity extension assays

As previously described by Lundberg, the expression of full-length SARS-CoV-2 spike protein and its subunits in HEK293 cells was further evaluated using highly sensitive PEA (Lundberg et al., 2011). A total of 30 μg of rabbit anti-SARS-CoV-2 (2019-nCoV) spike-RBD polyclonal antibody (Sinobiological, Cat. No. 40592-T62) was activated with dibenzyl cyclooctene-NHS ester (DBCO-NHS ester; Sigma-Aldrich) at room temperature (RT) for 30 min. Unreacted DBCO-NHS ester was removed using a 7 K molecular weight cut-off (MWCO) Zeba desalting spin column (Thermo Scientific). The activated antibody was split into two aliquots; each was incubated overnight at 4 °C with a distinct pair of azide-modified oligonucleotides (Zhao et al., 2022). PEA was performed by incubating 1.33 nM of each oligonucleotide-conjugated antibody pair with 1 μL (or diluted equivalents) of culture medium collected at 24, 48, and 72 h post-transfection (p.t.). Incubation was carried out for 1 h at 37 °C or overnight at 4 °C to allow proximity-based hybridization through specific binding to the S1 protein. Following incubation, 96 μL of an extension solution was added, comprising 1 × Hypernova buffer (BLIRT S.A.), 1.5 mM MgCl₂, 0.2 mM of each dNTP, 1 μM universal forward and reverse primers, 2 U/mL DNA polymerase (Invitrogen), and 5 U/mL Hypernova DNA polymerase. Extension and pre-amplification were performed at 50 °C for 20 min, followed by enzyme activation at 95 °C for 5 min, and 17 cycles of pre-PCR (95 °C for 30 s, 54 °C for 1 min, and 60 °C for 1 min). For quantitative real-time PCR (qPCR), 2.5 μL of the extension/pre-PCR product was transferred to a 96- or 384-well plate and mixed with 7.5 μL of qPCR mix. The qPCR mix included: 1 × PCR buffer (Invitrogen), 2.5 mM MgCl₂, 0.2 mM dNTPs, 1.6 μM ROX reference dye (Invitrogen), 0.3 μM molecular beacon (Biomers), 0.9 μM of each primer, 0.1 U Uracil N-glycosylase, and 0.5 U recombinant Taq polymerase (Invitrogen). The qPCR protocol include initial incubation at 25 °C for 30 min, denaturation at 95 °C for 5 min, followed by 30 amplification cycles (95 °C for 15 s, 60 °C for 1 min). All PCR primers and molecular beacon sequences were as described by Lundberg et al. (2011). Among the spike protein subunits, the RBS, SS-RBD, and S1 regions—as well as the full-length spike protein—were successfully detected. The S2 fragment was not detected in these assays.

### Statistical analysis

All statistical analyses were performed using GraphPad Prism software version 7.0 (GraphPad Software, San Diego, CA, United States). Differences between the two groups were assessed using unpaired two-tailed Student’s t-tests. Comparisons among multiple groups were evaluated using one-way analysis of variance (ANOVA). A *p*-value of < 0.05 was considered statistically significant.

## Results

### The spike constructs exhibited significantly higher expression in human embryonic kidney (HEK293) cells than in A549 lung adenocarcinoma epithelial cells

**Figure 1:**
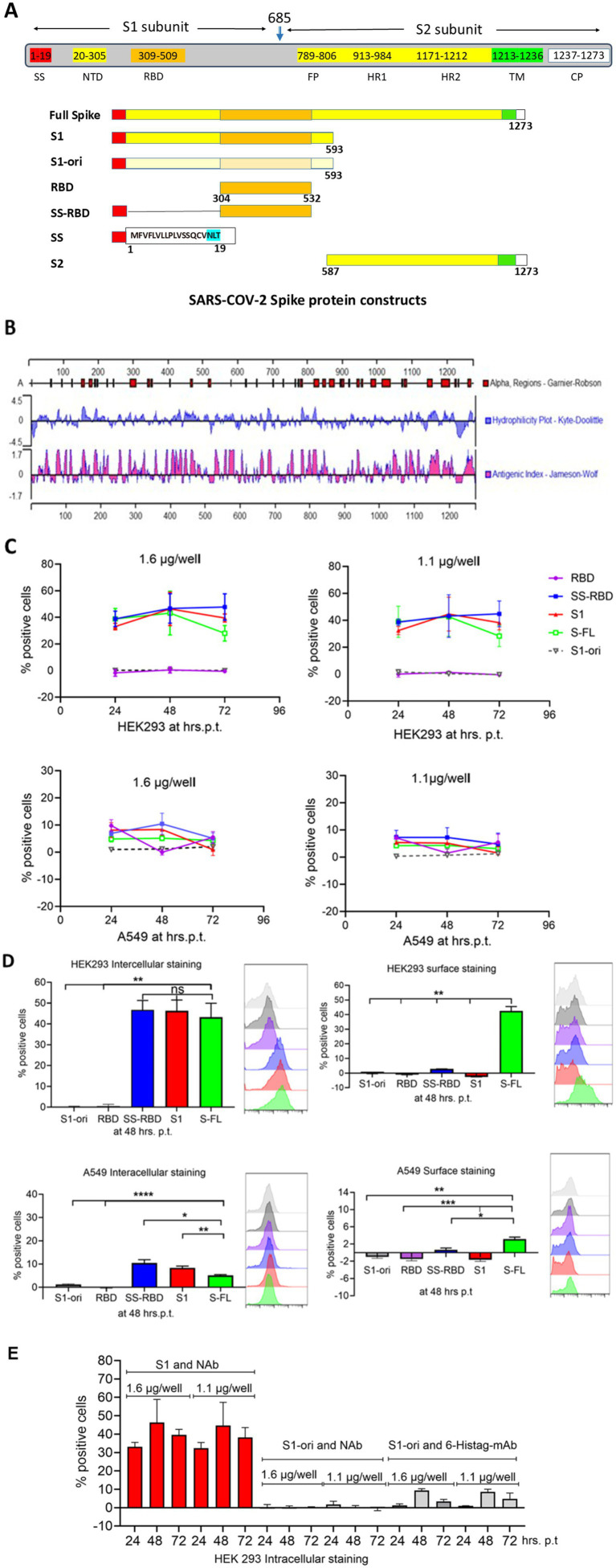
Characterization of SARS-CoV-2 spike protein constructs and their expression profiles in A549 and HEK293 cells. **(A)** Schematic representation of engineered spike protein domains: full-length spike (1-1273 aa), S1 (1-593 aa), RBD (304-532 aa), SS-RBD (N-terminal 19-aa signal peptide (SS) fused to RBD), and S2 domain (587-1,273 aa). The signal sequence (SS) comprises 19 amino acids, including the NTL (highlighted in blue), which represents a potential N-linked glycosylation site. The receptor binding motif (RBM) encompasses residues 437-508. The blue arrow bar indicates the furin cleavage site, located at the junction between the S1 and S2 subunits. Residue 685 (arginine, R685) is a critical component of the polybasic cleavage motif (RRAR, spanning residues 682-685). Cleavage occurs between R685 and S686 and is catalyzed by the host protease furin. All protein constructs contained a 6X His-tag at the C-terminus. **(B)** Bioinformatics predicts the full-length spike protein secondary structure, hydrophilicity and hydrophobicity, and antigenic epitopes. **(C)** Time-course expression analysis by FACS. Spike and fragment kinetic expression was detected by intracellular staining with a neutralization antibody (NAb) against the S1 subunit and empty vector FACS-based quantification. The empty pUC57 vector was used as a background control. Normal rabbit serum was used as an antibody control. **(D)** The expression levels of the full-length spike protein and subunits were evaluated by intracellular staining and cell surface staining with an NAb against the S1 subunit. **(E)** Kinetic expression of S1-ori was detected by anti 6-His mAB in FACS analysis and intracellular staining. S1-ori is the original sequence, and the S1 subunit is a humanized genomic sequence. All experiments were performed thrice, with duplicate samples in each. Error bars represent the mean ± SD. **p < 0.01; ***p < 0.001.

Based on the first reported SARS-CoV-2 genome (GenBank: MN908947.3), codon-optimized (GenScript) spike protein constructs were engineered in this study featuring C-terminal 6×His tags, including: full-length spike (1–1,273 aa), S1/RBD/SS-RBD fragments, S2 domain, and original S1 control (S1-ori), all cloned into pUC57 mammalian expression vectors containing CMV immediate-early promoter, SV40 polyA signal, and stop codon (Figure 1A). Critical design features included: (i) preservation of native furin cleavage site (RRAR↓S) and ectodomain architecture, (ii) incorporation of 19-aa signal peptide (except RBD/S2 constructs), and (iii) comprehensive in silico analysis predicting 78% hydrophilic/antigenic domains versus 22% hydrophobic regions (Figure 1B). The results revealed that the full-length spike protein contained numerous antigenic epitopes and hydrophilic domains, while hydrophobic regions were relatively limited.

Polyclonal NAb was used in the FACS analysis. Two plasmid doses were used (1.6 μg and 1.1 μg per well) in parallel to assess expression efficiency. The SS-RBD, S1 subunits, and full-length spike protein exhibited comparable expression levels, with approximately 40% of HEK293 cells testing positive at 24 h, p.t. (Figure 1C). Expression rates increased to 45–55% at 48 h p.t., with the SS-RBD fragment showing the highest expression at 72 h p.t. In contrast, the S1 fragment and full-length spike protein showed slightly reduced expression levels, with approximately 40% of cells positive. The S1-ori construct exhibited an expression rate of approximately 1% in FACS analysis. Overall, expression levels were higher in HEK293 cells than in A549 cells.

Subsequently, the surface expression of the transfected proteins was assessed. Among all constructs tested, the full-length spike protein demonstrated the highest surface expression in HEK293 and A549 cells. At 48 h p.t., approximately 40% of HEK293 cells expressed the spike protein on their surface (Figure 1D). The S1-ori construct exhibited a low expression rate in FACS analysis. To validate these findings, a monoclonal anti-6×His tag antibody was used to compare the expression of S1-ori to the codon-optimized S1 construct. Consistently, S1-ori demonstrated minimal expression in both assays, regardless of the antibody being used (Figure 1E). Based on these results, it was concluded that S1-ori is unsuitable for vaccine development. Notably, both plasmid doses (1.6 and 1.1 μg) yielded comparable FACS results. Therefore, the highest dose (1.6 μg/well) was selected for subsequent experiments. Consequently, all spike protein constructs used in subsequent experiments were codon-optimized for enhanced expression in human cells.

### Anti-6×His tag monoclonal antibody effectively detected RBD expression in transfected cells

**Figure 2:**
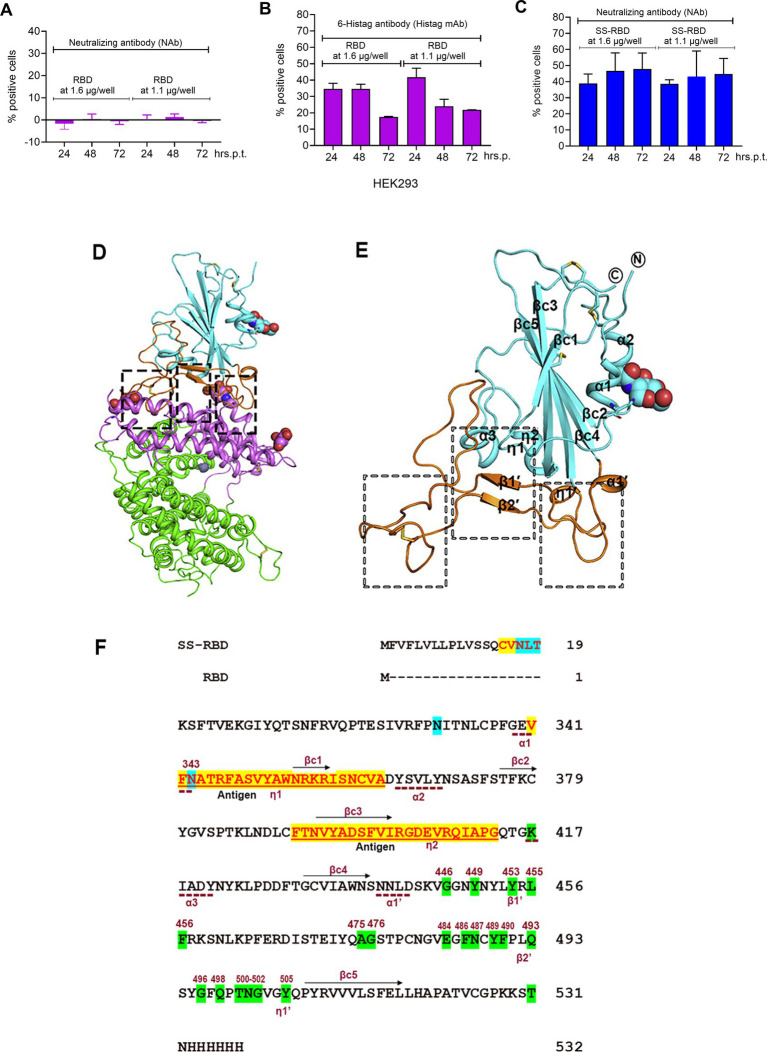
Structural and immunological characterization of RBD variants with ACE2 binding and antibody detection profiles. **(A)** Kinetics of RBD expression in HEK293 cells were detected by intracellular staining with an NAb against the S1 subunit at two plasmid doses (1,6 and 1,1 μg/well). The empty pUC57 vector was used as a negative control. **(B)** RBD expression kinetics were assessed by FACS using an anti-6His-tag mAb. **(C)** Kinetics of SS-RBD expression were detected by intracellular staining by NAb against the S1 subunit. **(D)** The structure model of the SARS-CoV-2 RBD bound to hACE2. The core and external subdomains in the SARS-CoV-2 RBD are colored cyan and orange, respectively. Human ACE2 subdomains I and II are colored violet and green, respectively. The contact sites are further delineated in **(E)**. **(E)** A representation of the SARS-CoV-2 RBD structure. Key contact sites of the amino acids are marked according to Wang et al. (2020). **(F)** The initial signal peptide sequence (SS) consists of 19 amino acids. The cleavage site is located at amino acid 13. A partial immunogenic epitope in the SS is marked in red letters. The glycan site “NLT” or “N” is highlighted in blue—structure-based sequence alignments of the RBD related to **(D,E)**. Secondary structure elements are defined using the ESPript algorithm (Robert and Gouet, 2014) and labeled according to the RBD structure reported in the study. The dashed lines represent *α* strands, and the arrows represent *β* strands. Three glycosylation sites (N) are highlighted in blue. Two antigenic epitopes, η1 and η2, are highlighted in yellow. Critical RBDs consisting of essential amino acids are highlighted in green, and the number of positions of amino acids is shown at the top.

FACS using NAb failed to detect the RBD subunit in transfected cells (Figure 2A), suggesting that the RBD domain was either not exposed for NAb recognition or masked as a cryptic epitope. To test this, the NAb was replaced with an anti-6×His monoclonal antibody, which successfully detected RBD expression (Figure 2B). Interestingly, the SS-RBD fragment was also detected in both antibodies’ assays (Figure 2C). To better understand the interaction between RBD and ACE2, the three-dimensional structure of the RBD-ACE2 complex was referenced (Wang et al., 2020). The presence of the signal sequence (SS) appeared to influence the conformation of upstream RBD epitopes, thereby facilitating exposure of the α1 epitope and leading to recognition by NAb (Figures 2D,E). Taken together, these findings suggest that ACE2 binding masks the antigenicity of the RBD domain in host cells, a critical consideration for vaccine design. Critical amino acid residues involved in this interaction are highlighted in Figure 2F.

### The SS domain positively influences the secretion and expression of the RBD subunit

**Figure 3:**
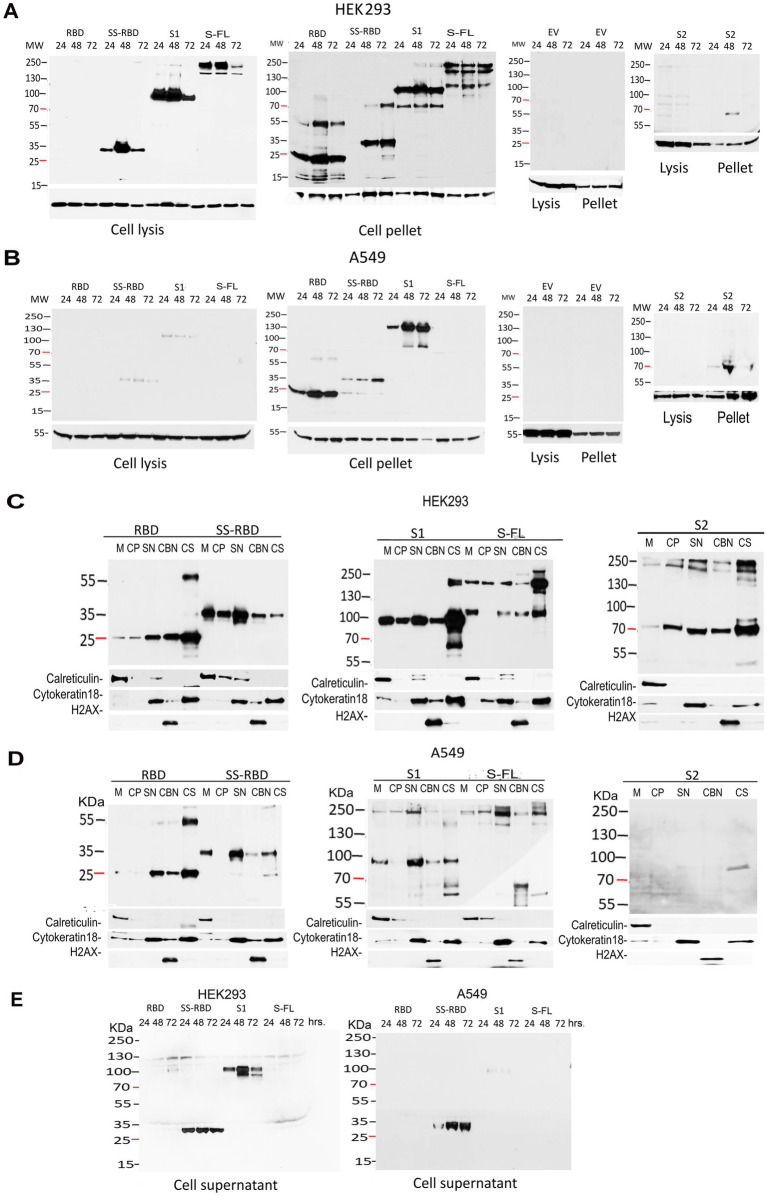
Spatiotemporal expression profiling of SARS-CoV-2 spike variants in mammalian cells. **(A)** Kinetic expression of full-length spike protein and subunits in cell lysates, cell pellets, and subcellular fractions detected by immunoblotting. EV means empty vector. **(B)** Western blot detection of spike protein subunits using an anti-His antibody. RBD (26 kDa), SS-RBD, S1 subunit (100 kDa), and S-FL (150 kDa). Film exposure for 1 min. An anti-tubulin antibody that detected tubulin was used as an internal reference in cell lysate and pellet samples. **(C,D)** Immunoblot assay results of SARS-CoV-2 full-length spike protein and subunits in subcellular fractionated materials. The Subcellular Protein Fractionation Kit fractionates cells from HEK293 **(C)** or A549 **(D)**. Normalized concentrations of each extract (10 μg) (the cytoskeleton extract concentration was 5 μg) were analyzed by WB using specific antibodies against proteins from various cellular compartments, including calreticulin for detecting the membrane (M) and endoplasmic reticulum (SN) fraction and cytoskeleton (CS) fraction. The soluble nuclear (SN) fraction was detected using a CS marker (Cytokeratin 18), and the chromatin-bound nuclear (CBN) fraction was detected using a rabbit anti-histone H2AX mAb. A rabbit anti-His tag mAb detected the levels of the spike protein and subunits. Cell supernatants were harvested, denatured, and electrophoresed on mini-Nu-PAGE gels. The protein bands were visualized for 15 min in HEK293 cells and 2 h in A549 cells. **(E)** Kinetic expression of SARS-CoV-2 spike protein full-length (S-FL) and subunits in cell culture supernatants from HEK293 and A549 cells by immunoblotting.

Next, the expression of the spike protein and subunit was analyzed by SDS-PAGE and immunoblotting using an anti-6×His-tag monoclonal antibody. The same plasmid DNA constructs exhibited relatively higher expression in HEK293 cells compared to A549 cells. Each spike protein fragment displayed distinct expression characteristics and subcellular localization. SS-RBD and S1 subunits exhibited stronger expression than RBD and the full-length spike protein in cell lysates at 24, 48, and 72 h p.t. (Figures 3A,B). In the cell pellet fraction, RBD, SS-RBD, and S1 accumulated significantly, whereas expression of the full-length spike protein was lower than that of the subunits (Figures 3A,B). Although the S2 fragment was also detected in the cell pellet, it exhibited the lowest expression among all constructs in both cell lines (Figures 3A,B). No specific bands were detected in the vector control group. Interestingly, the full-length spike protein appeared as multiple bands of varying molecular weights, suggesting ongoing post-translational modifications, such as glycosylation or conformational processing, during intracellular trafficking. These results demonstrate a critical role for the SS domain in optimizing RBD production efficiency.

### PEA revealed logarithmic-scale differences in the expression levels of the spike protein and its subunits

**Figure 4:**
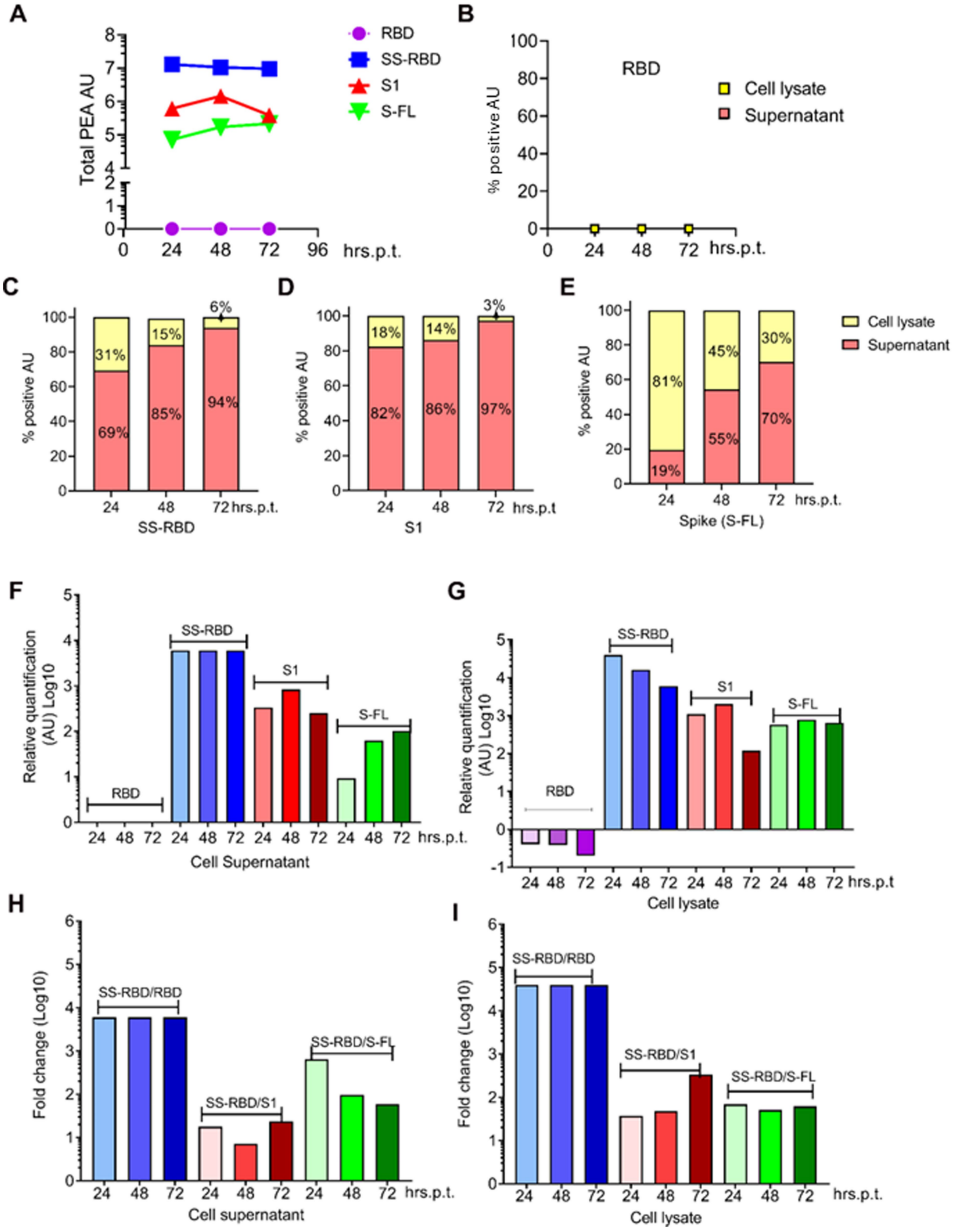
Quantitative profiling of spike variant expression dynamics by PEA. **(A)** Total PEA relative quantification of activation for spike proteins in cell supernatants and cell lysates from HEK293 cells. The sample volume of harvested cell lysis or cell supernatant is presented in Materials and Methods. **(B)** PEA relative quantification in the RBD domain. RBD exhibits undetectable PEA activity unit (AU) due to localization in cell pellets. **(C–E)** Total PEA relative quantification activation as an AU for SS-RBD, S1 subunit, and the full-length spike protein, respectively. SS-RBD showed the highest expression (10^7^), the S1 subunit showed middle-level expression (10^6^), and the full-length spike protein showed low-level expression (approximately 10^5^). Not shown: RBD was undetectable in the PEA because it was only expressed in the cell pellets. **(F,G)** Comparison of the expression of the full-length spike protein and subunits in cell supernatants and cell lysates, respectively. **(H,I)** Fold change in SS-RBD expression over the expression of RBD, S1 subunit, and a full-length spike protein in cell supernatants and lysates. At each point, duplicate samples were collected and analyzed using PEA.

The PEA method consists of three main steps: a liquid antigen brings oligonucleotide-conjugated antibody molecules into proximity, extension/pre-PCR, and readout via real-time PCR. The reaction used 1 μL of undiluted or 10-fold diluted samples. The antigen was measured up to 7 logs for SS-RBD, 6 for S1, and 5 for the full-length spike protein. Thus, PEA is sensitive, stable, and has a broad dynamic range for RBD antigen detection. The full-length spike protein was undetectable in immunoblot but it could be detected by PEA assay at level 10–100 activity units (AU), demonstrating that PEA was superior to immunoblot for antigen detection (Figure 4A). PEA quantification showed a 100- or 10-fold increase in SS-RBD or S1 levels compared to full-length spike protein levels (Figure 4A). However, RBD without the SS was not detected with the NAb in either the immunoblot or PEA method (Figure 4B). PEA analysis of total expression, measured in both cell lysates and supernatants, was normalized by multiplying the measured expression by the volume obtained for each sample. Furthermore, the study analyzed the proportion of intracellular versus extracellular distribution. The results showed that 69–86% of SS-RBD and S1 were secreted into the cell supernatant at 24–48 h post-transfection (p.t.), increasing to 94–97% at 72 h p.t. (Figures 4C,D). In contrast, the full-length spike protein showed supernatant expression levels of 19, 55, and 70% at the respective time points, with consistently high expression retained in the cell lysate (Figure 4E).

The SS stimulated the movement of its peptide from the cytoskeleton toward the cell surface until it was released into the cell supernatant (Figures 4F–H). The PEA demonstrated that SS-RBD expression is superior to the S1 fragment and the full-length spike protein in the HEK293 supernatant (Figure 4A). RBD expression was lower than the standard curve range at three time points, so it was undetectable and consistent with the above FACS data (Figures 4B,C). Notably, two different Nabs negatively detected RBD antigens in the PEA and FACS experiments. However, RBD expression was negative in both assays. The results of this study showed that completely blocked RBD antigenicity was undetectable (Figures 4B,C). Notably, the cell supernatant from HEK293 cells transfected with RBD was negative (Figure 4B). At the same time, the full-length spike protein remained detectable even at a 10-fold dilution. Total S1 expression ranged between 10^5^ and 10^6^ arbitrary units (AU) across the time course, with S1 levels approximately one log higher than those of the full-length spike protein. SS-RBD showed the highest expression among all constructs, reaching up to 10^7^ AU, which was approximately 100-fold higher than the full-length spike protein in HEK293 cells (Figures 4A,F–I; Table 1).

**Table 1:** Comparative expression, secretion, and characterization of the SARS-CoV-2 full-length spike protein and its subunits in mammalian cells.

Samples	Extracellular distribution	Cell surface	Intracellular distribution	Nucleus	Predominant location	PEA arbitrary units (AU) in total
RBD	-	UD	++	−	Cytoskeleton	Negative
SS-RBD	++++	+/−	++	−	Extracellular distribution	10^7^ AU PEA
S1	++	+/−	++	+	Extracellular distribution	10^6^ AU PEA
Spike (S-FL)	+	+++	+	+	Cell surface	10^5^ AU PEA
S2	UD	UD	+	−	Cytoskeleton	UD

The expression levels of the full-length spike protein and its subunits were calculated. 1. PEA relative quantification and immunoblot assay calculated the expression level of the full-length spike protein and its subunits in the cell supernatant. 2. FACS analysis, WB, and confocal microscopy were used to determine the cell surface expression of the full-length spike protein and subunits. 3. FACS analysis, PEA, WB, and confocal microscopy were used to determine the intracellular expression of the full-length spike protein and subunits. 4. The expression levels of the full-length spike protein and subunits in the cell nucleus were evaluated by confocal microscopy. UD means undetected. “++++” means high expression; “+++” means relatively high; “++” means average expression; and “+” means low expression; “+/−” means hardly detectable; “-” means undetectable expression. The PEA assay showed that the total expression of each subunit, measured in cell lysates and supernatants, was reported in arbitrary units (AU).

### Confocal microscopy revealed that both the S1 subunit and the full-length spike protein were localized predominantly in the cytoplasm and partially in the nucleus of transfected cells

**Figure 5:**
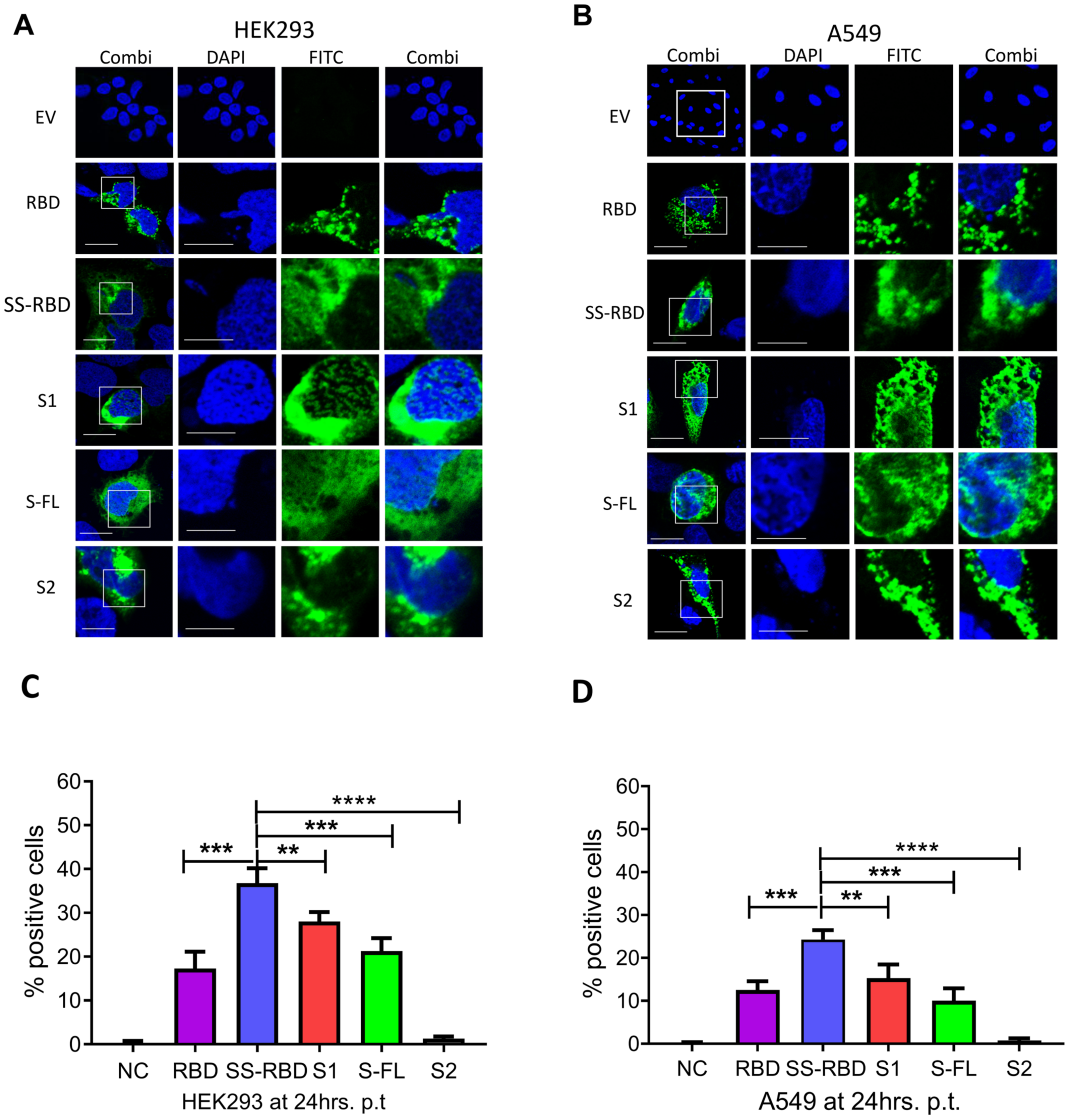
Subcellular localization and expression efficiency of full-length spike protein (S-FL) and its subunits in transfected mammalian cells. **(A,B)** HEK293 and A549 cells were grown on glass coverslips overnight; the cells were transfected with plasmid DNA, incubated for 24 h, fixed, and processed for IF staining (Materials and Methods). The S-FL and subunits’ subcellular distribution was stained with an anti-6-his tag mAb and FITC secondary Ab (green), and nuclei were stained with DAPI (blue). Bar: 10 μm. **(C,D)** HEK293 and A549 cells were also quantified as the percentage of cells positively transfected with the S-FL and subunits by IF microscopy. The graph presents the averages of triplicate samples. The asterisks indicate significant differences, as demonstrated by an unpaired t-test (*p < 0.05; **p < 0.01; ***p < 0.001); ns, not significant. The data are presented as the mean ± SE from three samples.

A confocal microscope was used to assess the expression and subcellular localization of the spike protein and its subunits in HEK293 and A549 cells. At 24 h p.t., cells were immunostained to visualize protein distribution. The RBD, SS-RBD, and S2 subunits were predominantly localized in the cytoplasm, whereas the S1 fragment and full-length spike protein were detected in both the cytoplasm and nucleus. No fluorescence was observed in non-transfected cells or those transfected with empty vectors, confirming the specificity of the staining. The subcellular distribution patterns of S1 and full-length spike protein were consistent across both HEK293 and A549 cell lines (Figures 5A,B). The proportion of positively stained cells was quantified by standard fluorescence microscopy, with counts performed in at least four separate fields. Among the constructs analyzed, SS-RBD exhibited the highest expression levels, followed by mid-range expression of RBD, S1, and full-length spike protein, while S2 displayed the lowest expression (Figures 5C,D). These findings complement the fractionation data of this study (Figure 3) and visually confirm the different localizations among the spike protein variants.

## Discussion

This study systematically examined the expression, antigenicity, and subcellular localization of SARS-CoV-2 spike protein constructs and their subdomains using complementary methods (PEA, FACS, WB, and IF) in HEK293 and A549 cells. The results of this study show that construct design—especially codon optimization and SS addition—significantly influences protein expression, intracellular trafficking, secretion efficiency, and epitope availability. Consistent with prior studies, HEK293 cells showed higher expression levels than A549 cells, likely due to differences in transfection efficiency and secretory ability. Additionally, codon optimization was crucial for effective expression, as the original S1 construct (S1-ori) produced minimal protein. These findings emphasize the importance of optimizing both host cell systems and gene sequences for recombinant antigen production.

The study observes that RBD was efficiently expressed but predominantly localized within the cytoplasm, where it was closely associated with CS structures. In contrast, substantial amounts of SS-RBD and the S1 subunit were detected in the extracellular supernatant, indicating efficient secretion. Western blot analysis further confirmed the robust expression of SS-RBD across multiple cellular fractions. At the same time, PEA quantification revealed that SS-RBD expression was approximately two orders of magnitude higher than that of the full-length spike protein. These findings suggest that SS-RBD may yield higher antigen levels and potentially enhanced immunogenicity compared to larger spike constructs.

SS plays a central role in these differences. This study extends the native N-terminal signal peptide to 19 amino acids and introduces an additional glycosylation site at asparagine 17 (N17), which overlaps with a known neutralizing epitope (Cerutti et al., 2021). This design appears to enhance protein targeting to the secretory pathway and may improve folding or stability. From an immunological perspective, effective antigens must preserve conformational epitopes while remaining structurally accessible. Notably, SS-RBD fulfills these criteria and may form particulate or multimeric structures, which are known to enhance immune recognition and antigen presentation.

In contrast, RBD and S2 constructs lacking the SS failed to enter the secretory pathway efficiently and instead accumulated intracellularly, co-localizing with CS and endoplasmic reticulum (ER)-associated compartments. This highlights the essential role of SS in directing proper intracellular trafficking and secretion. Moreover, the observation that NAbs failed to detect RBD in certain assays suggests that key epitopes may be conformationally masked in the cellular context, potentially due to ACE2 interactions or structural rearrangements. This finding underscores the importance of considering epitope accessibility—not just expression level—in antigen design. In addition, previous studies have shown that NAbs can target regions outside the RBD (e.g., S309 and N343 glycan-associated epitopes), further supporting a broader perspective on spike antigen design (Fang et al., 2022; VanBlargan et al., 2022).

An intriguing finding of this study is the nuclear localization of the full-length spike protein and, to a lesser extent, the S1 subunit. Computational predictions indicate multiple putative nuclear localization signals (NLSs), particularly within the S1 domain, that may facilitate nuclear translocation. Consistent with this, confocal microscopy revealed accumulation of S1 both around and within the nucleus. These observations align with recent reports demonstrating that spike-derived proteins can enter the nucleus via nuclear pore complexes (NPCs) (Sattar et al., 2023).

The potential involvement of annulate lamellae (AL) structures further supports this mechanism (Eymieux et al., 2021). ALs are cytoplasmic membrane arrays enriched in NPC-like structures and are often observed in virus-infected cells (Cardinali et al., 1998). They may serve as reservoirs or intermediates for viral protein trafficking. Similar mechanisms have been described in other viral systems, including herpesviruses and hepatitis C viruses, in which NPC-associated pathways facilitate intracellular transport (Neufeldt et al., 2013). While the functional consequences of spike protein nuclear localization remain unclear, this phenomenon may have implications for host cell regulation, antigen processing, or immune modulation and warrants further investigation (Sattar et al., 2023).

From a vaccine development perspective, the findings of the study highlight important trade-offs between antigen size, expression efficiency, and immunogenicity. While the full-length spike protein is efficiently presented on the cell surface, its large size and complex post-translational processing limit overall yield. In contrast, smaller subunits, such as S1 and especially SS-RBD, are produced and secreted more efficiently, increasing their availability for immune recognition. Removal of the TM may further enhance antigen release, potentially improving systemic exposure and immune activation.

The RBD is a critical immunogen capable of eliciting both humoral and cellular immune responses. CD4^+^ T cells recognize RBD-derived epitopes and support B cell maturation and antibody production, often driving a Th1-biased response characterized by IFN-*γ* and TNF-*α* secretion (Moss, 2022). In addition, RBD-derived peptides can be presented via MHC class I molecules to activate CD8^+^ cytotoxic T lymphocytes, contributing to viral clearance (Khoo et al., 2023; Phan et al., 2023; Carretero et al., 2024). These properties make RBD-based constructs attractive vaccine candidates. The data in this study suggest that a soluble, signal sequence-enhanced RBD (SS-RBD) may offer advantages in expression, secretion, and antigen specificity, while potentially reducing off-target immune responses.

So far, subunit vaccines based on recombinant viral proteins have a well-established safety and efficacy profile. Examples include hepatitis B (HBsAg), varicella-zoster (Shingrix), and influenza (Flublok) vaccines, all of which rely on recombinant antigen production. These platforms avoid the use of live viruses, allow precise targeting of neutralizing epitopes, and support scalable manufacturing. In this context, the engineered spike subunits in this study, particularly SS-RBD, represent promising candidates for further development in both vaccine and diagnostic applications. SARS-CoV-2 vaccines remain essential for preventing severe disease, hospitalization, and mortality despite the continued evolution of SARS-CoV-2 variants. Recent vaccine strategies have focused on updating the antigen composition to match circulating Omicron-descendant lineages, including JN.1, KP.2, LP.8.1, and the emerging XFG variant. Regulatory agencies such as WHO, FDA, and EMA now recommend adaptive vaccine updates based on global surveillance data (Hao et al., 2024; Koolaparambil Mukesh et al., 2024; Alhumaid et al., 2026).

Finally, this study emphasizes the importance of selecting appropriate expression systems and optimizing antigen design for large-scale production (Keating and Noble, 2003; King et al., 2009; Pan et al., 2022). HEK293 cells consistently outperformed A549 cells in protein expression, supporting their use in recombinant antigen manufacturing. Among all constructs tested, SS-RBD exhibited the highest yield, approximately 10-fold greater than S1 and substantially higher than full-length spike protein, while maintaining high specificity.

Future studies should focus on structural characterization of SS-RBD to confirm preservation of key neutralizing epitopes and to guide further optimization. Stabilization of its three-dimensional conformation may enhance immunogenicity and enable dose-sparing strategies, ultimately improving vaccine efficacy.

In conclusion, the results of this study provide a comprehensive analysis of SARS-CoV-2 spike protein subunits, demonstrating that signal sequence engineering and construct design are critical determinants of antigen expression, secretion, and immunogenicity. The superior performance of SS-RBD highlights its promise as a next-generation vaccine antigen and provides a framework for the rational design of optimized spike-based immunogens.

## Data Availability

The raw data supporting the conclusions of this article will be made available by the authors, without undue reservation.

## Footnotes

1
www.biorad.com/webroot/web/pdf/lsr/literature/Bulletin_6376.pdf

## Author’s note

In this article, Figures 2D,E are adapted from an imperative article published by Wang et al. (2020).
